# Supplementary figures and images for: The inflammasome adaptor protein ASC promotes amyloid deposition in cryopyrin-associated periodic syndromes
Source: EMBO Mol Med. 2024 Dec 5;17(1):41–53. doi: 10.1038/s44321-024-00176-1 (PMC11731034; doi:10.1038/s44321-024-00176-1)

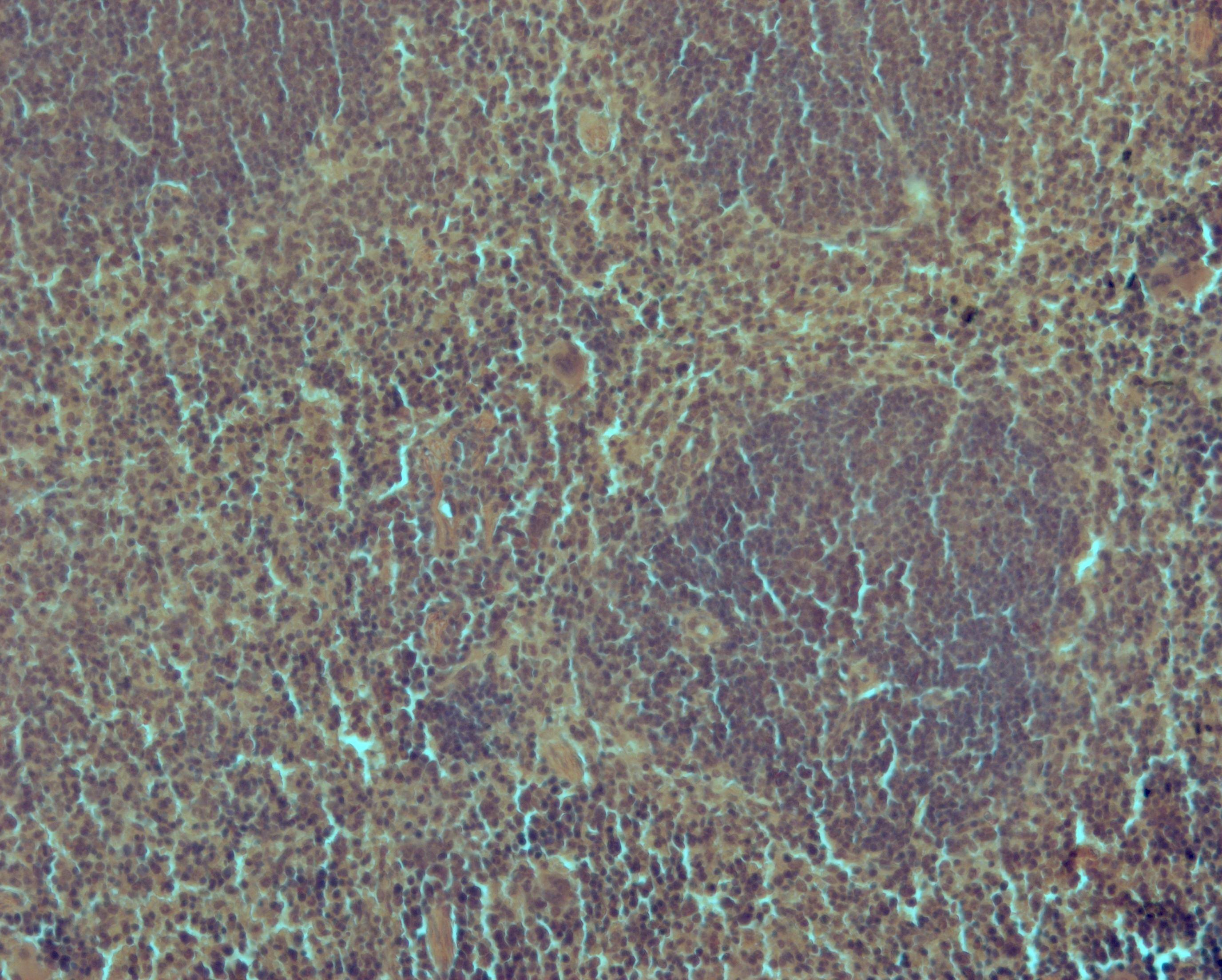

Supplement: Supplementary file 2 — Source data Fig. 1 [file 44321_2024_176_MOESM2_ESM.zip › Figure 1/1G/06 PYCARD KO SPLEEN.tif]

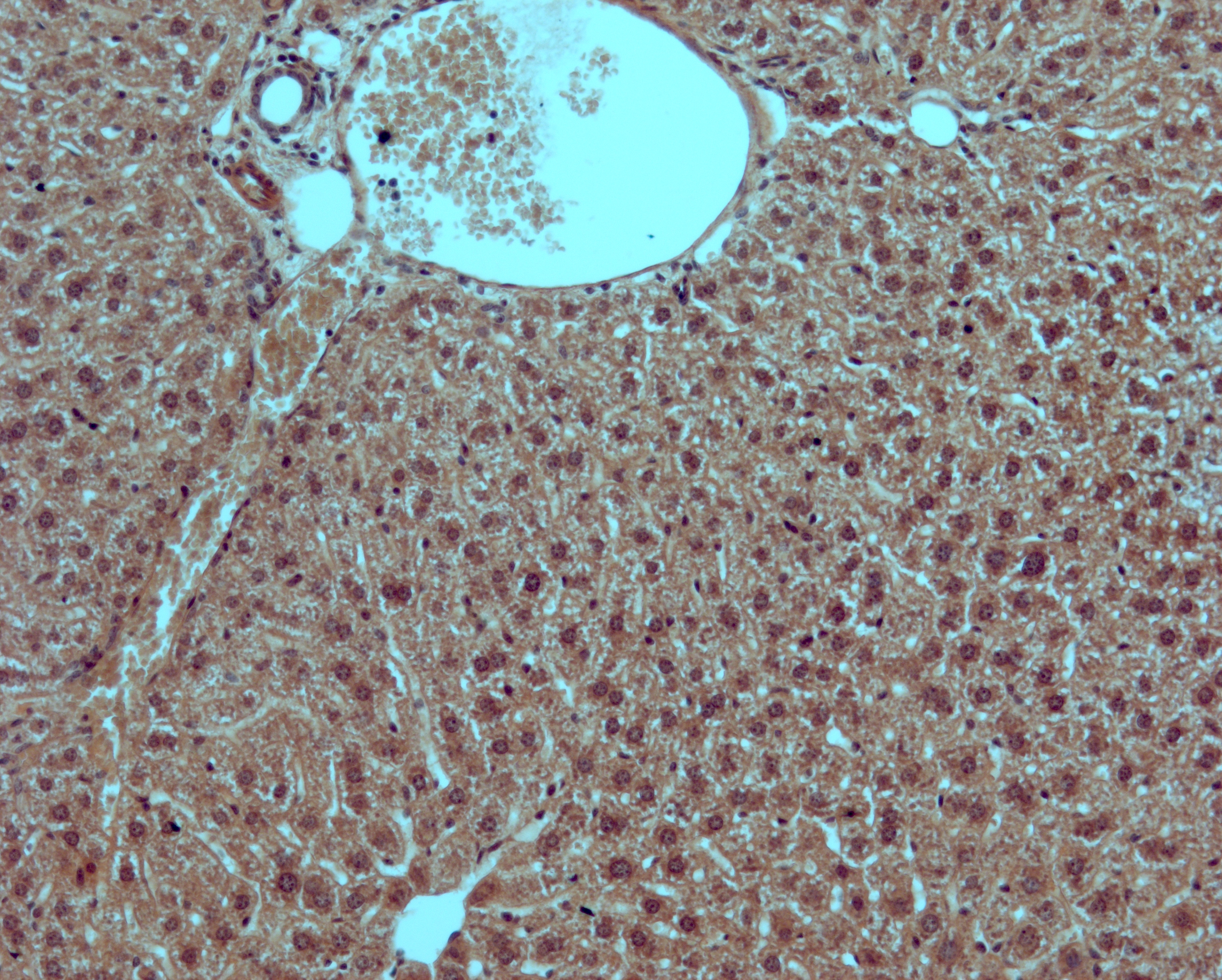

Supplement: Supplementary file 2 — Source data Fig. 1 [file 44321_2024_176_MOESM2_ESM.zip › Figure 1/1G/04 PYCARD KO LIVER.tif]

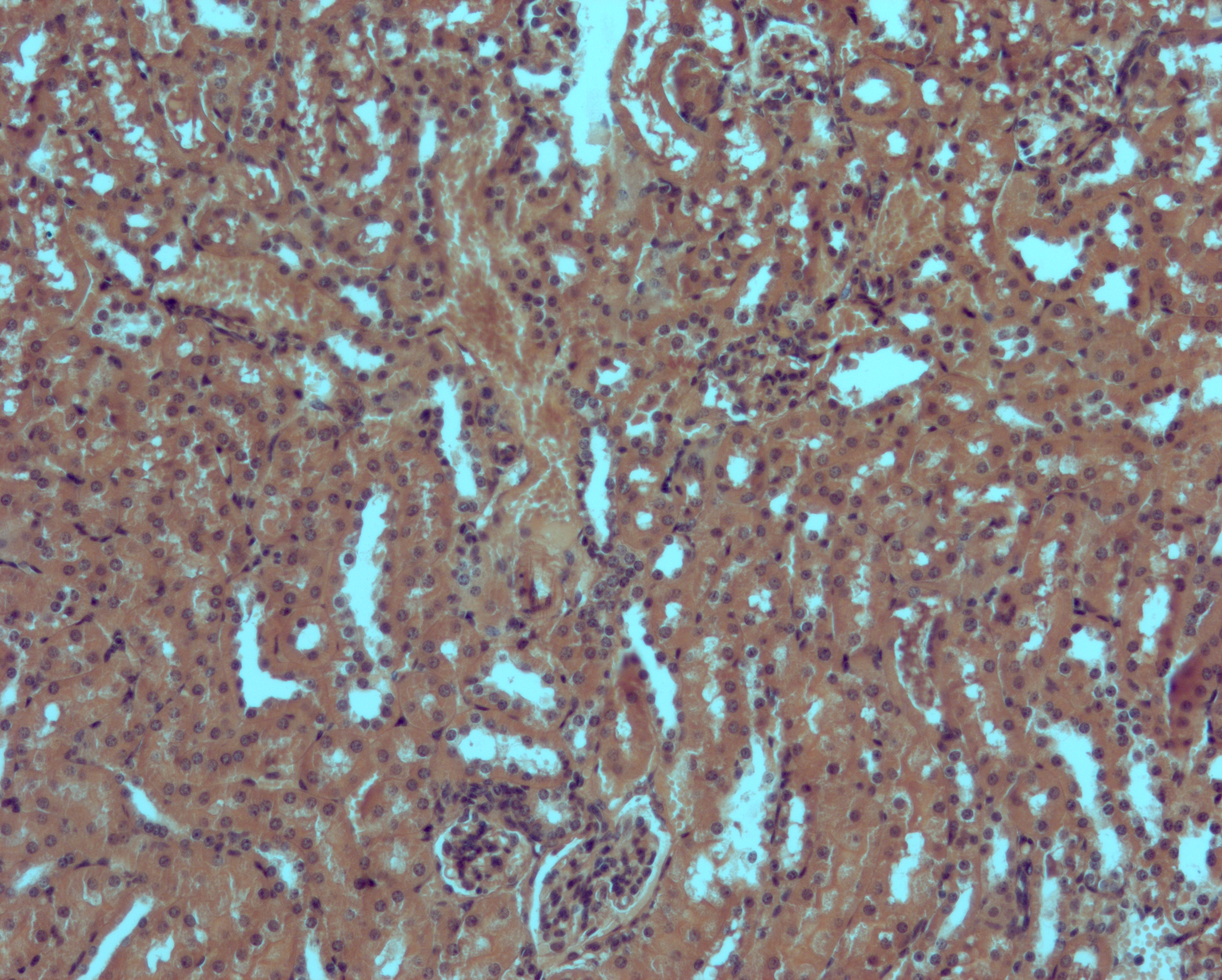

Supplement: Supplementary file 2 — Source data Fig. 1 [file 44321_2024_176_MOESM2_ESM.zip › Figure 1/1G/05 PYCARD KO KIDNEY.tif]

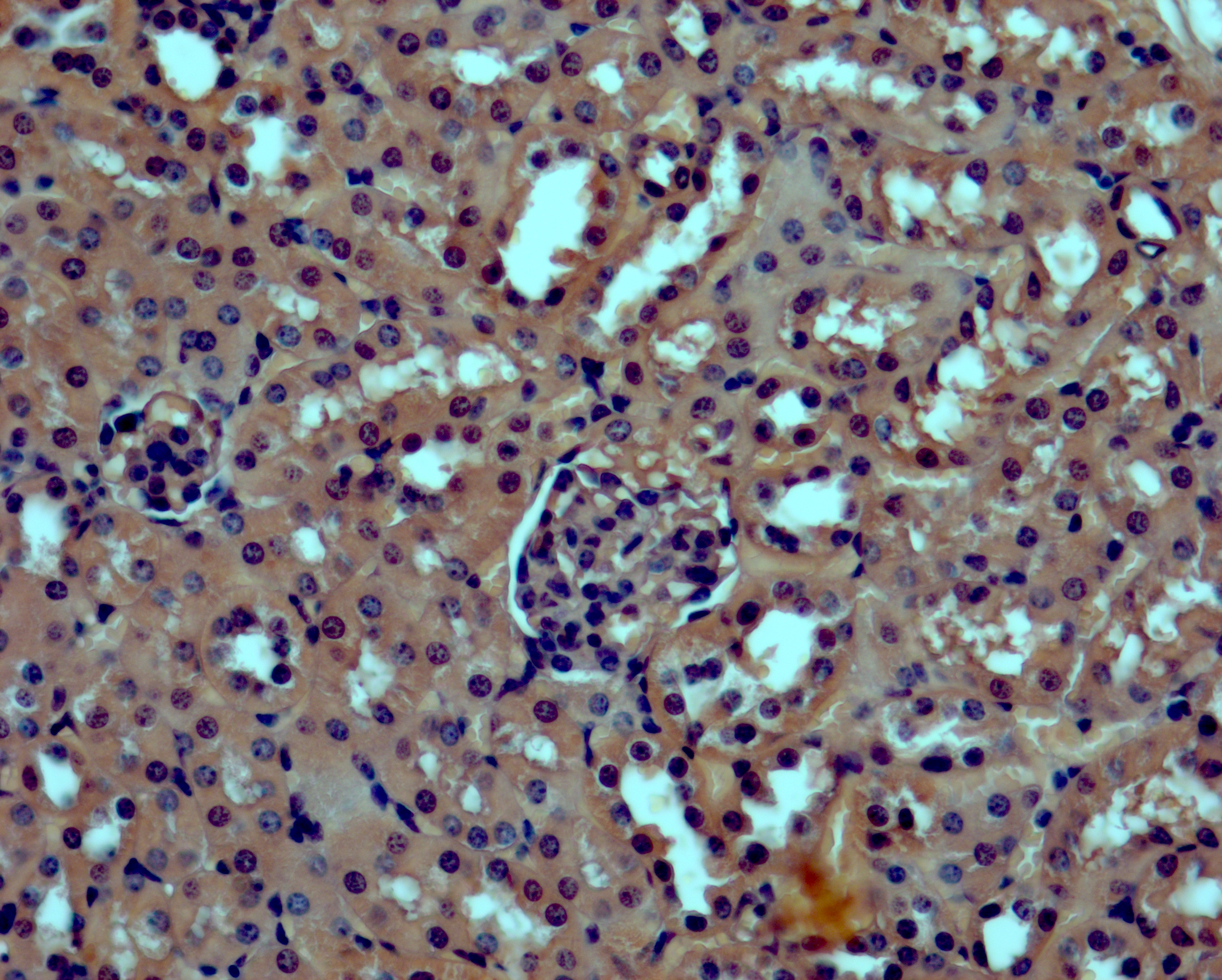

Supplement: Supplementary file 2 — Source data Fig. 1 [file 44321_2024_176_MOESM2_ESM.zip › Figure 1/1G/02 KIDNEY C57.tif]

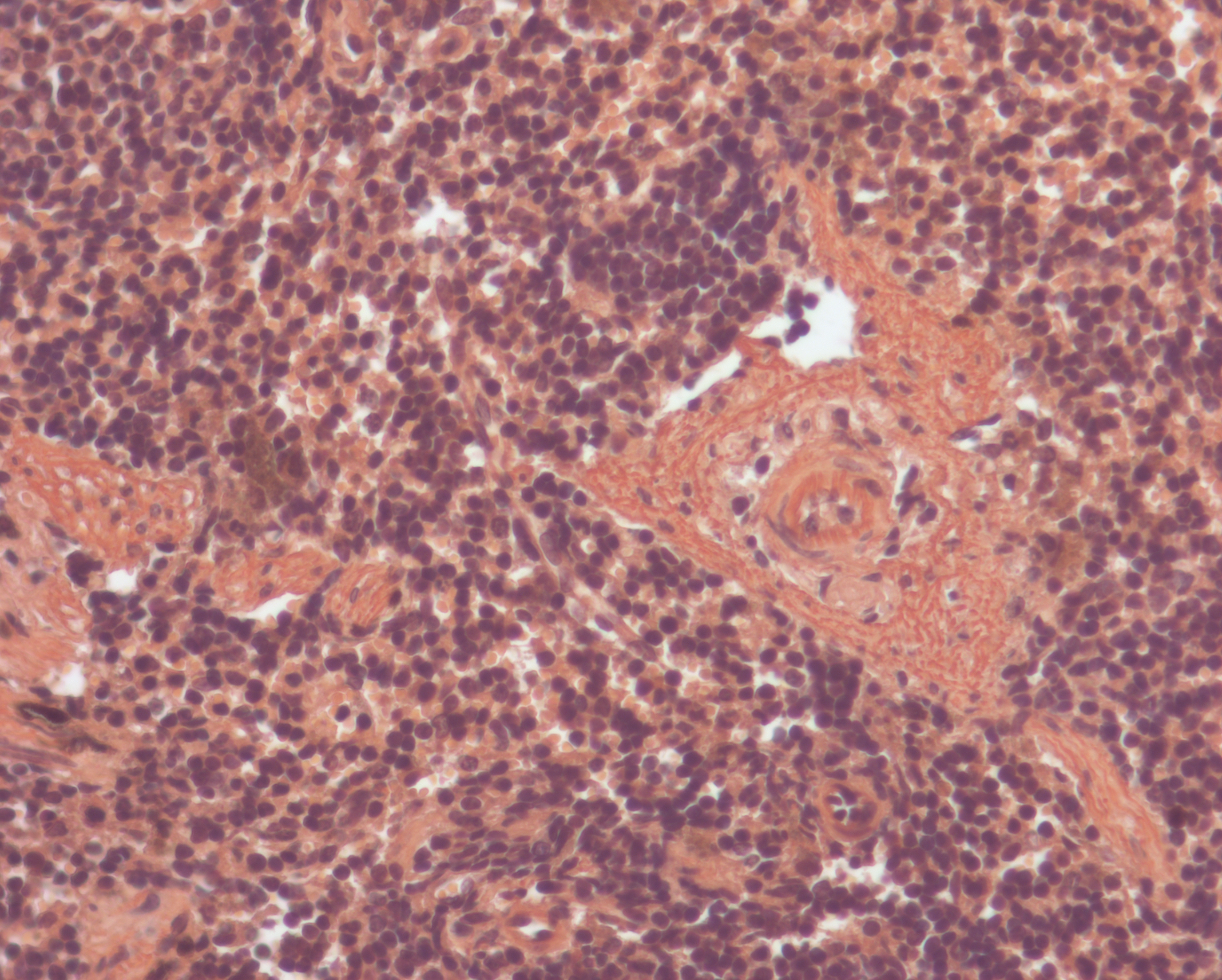

Supplement: Supplementary file 2 — Source data Fig. 1 [file 44321_2024_176_MOESM2_ESM.zip › Figure 1/1G/03 SPLEEN C57.tif]

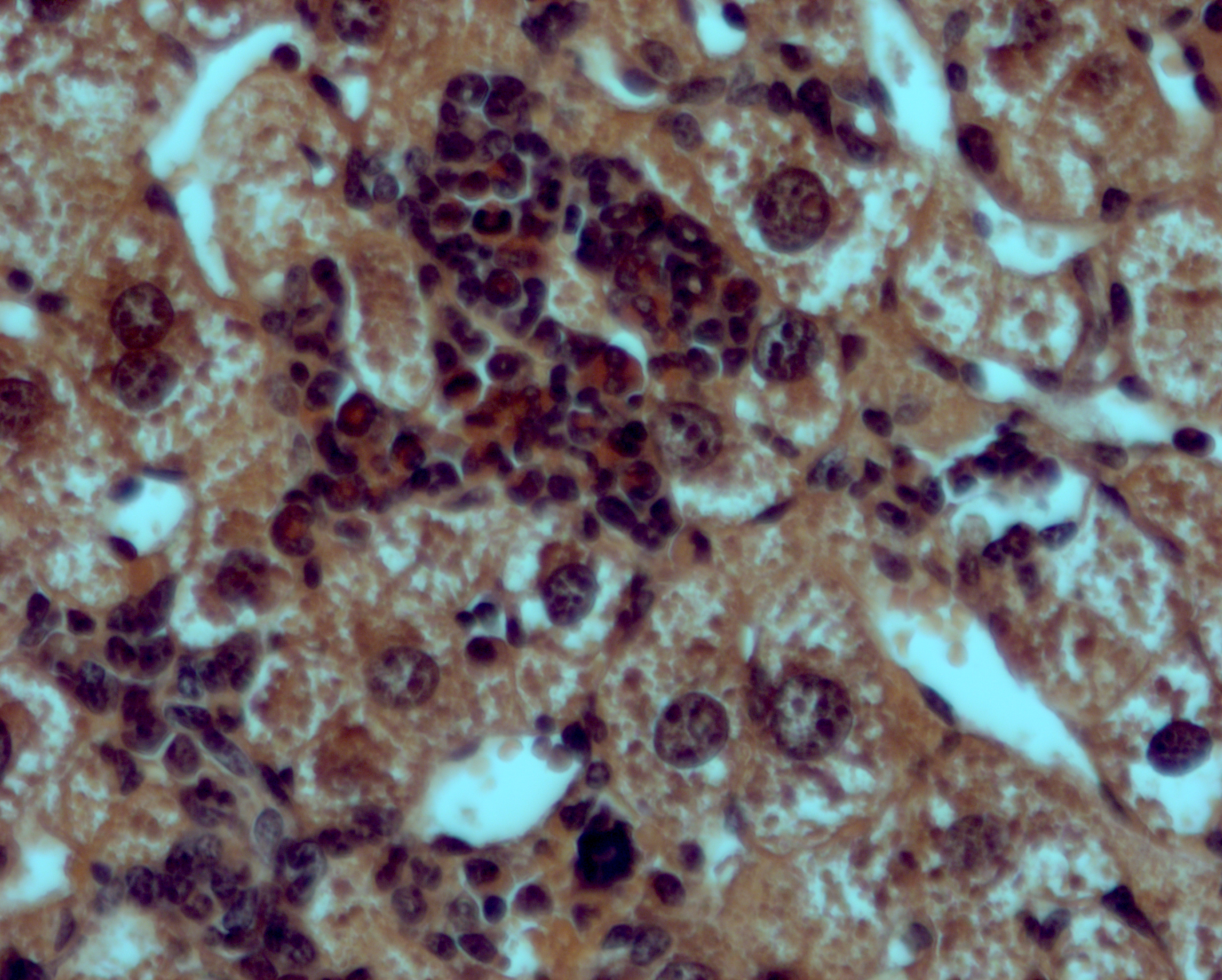

Supplement: Supplementary file 2 — Source data Fig. 1 [file 44321_2024_176_MOESM2_ESM.zip › Figure 1/1G/01 LIVER C57.tif]

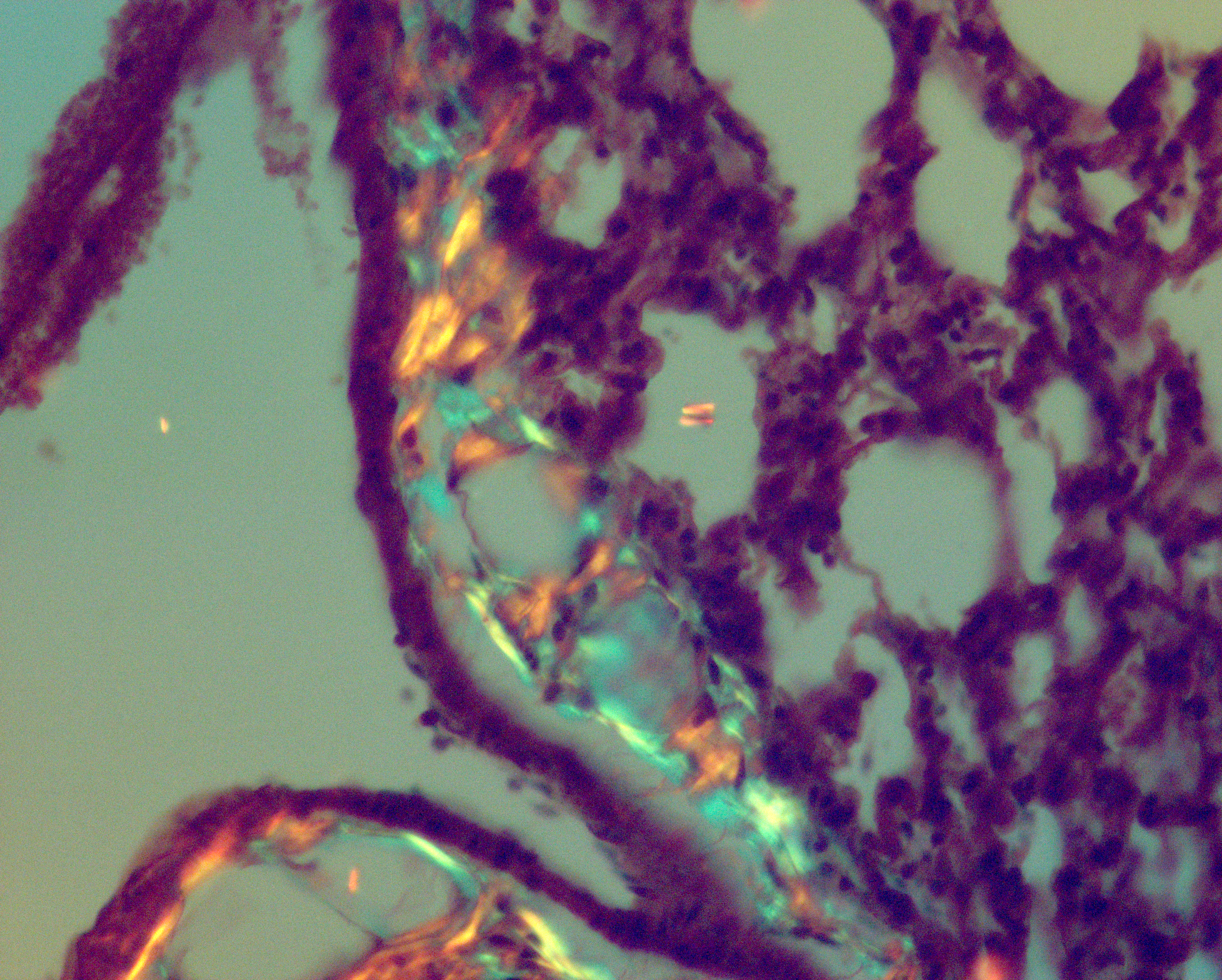

Supplement: Supplementary file 2 — Source data Fig. 1 [file 44321_2024_176_MOESM2_ESM.zip › Figure 1/1K/18D LUNG.tif]

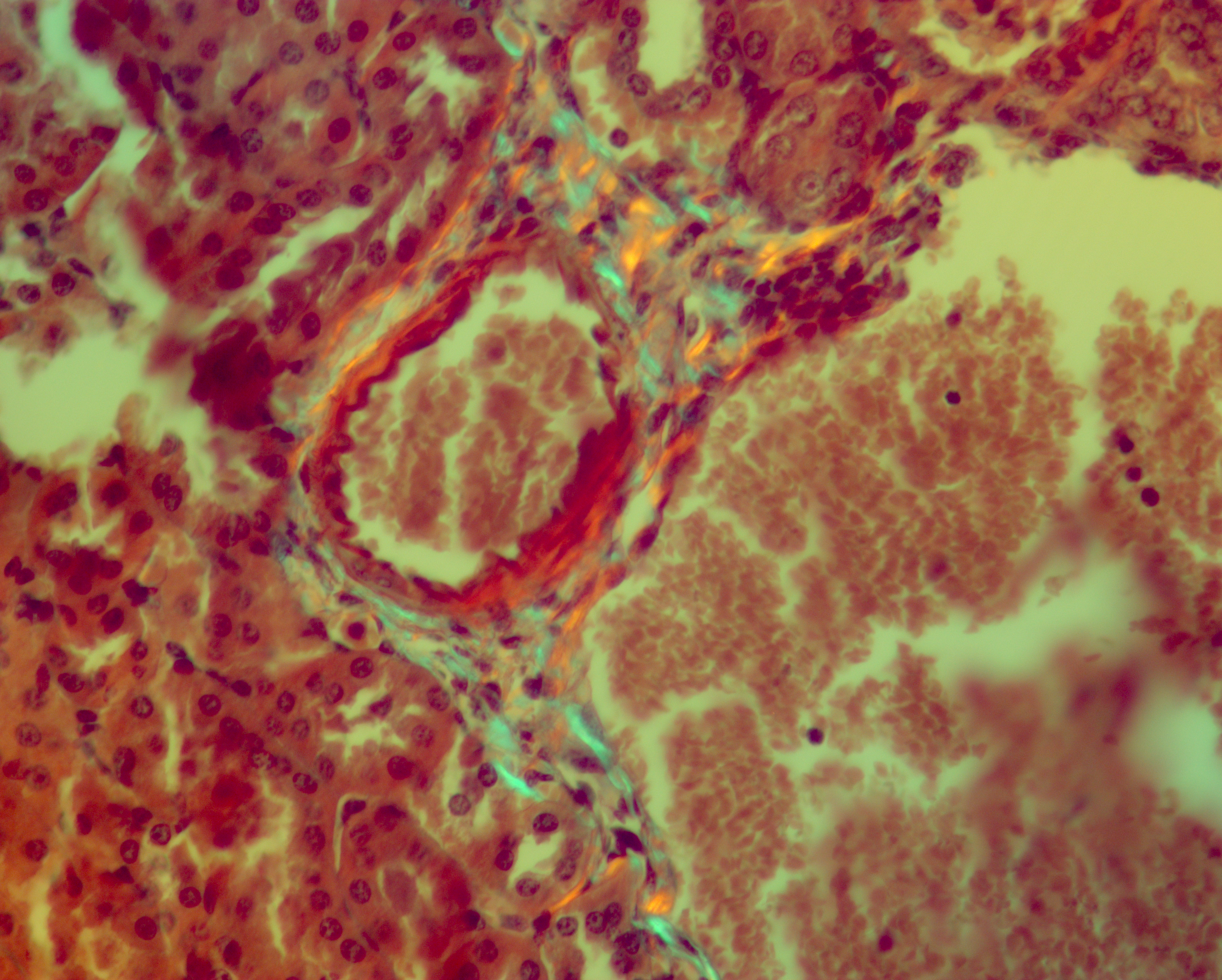

Supplement: Supplementary file 2 — Source data Fig. 1 [file 44321_2024_176_MOESM2_ESM.zip › Figure 1/1K/18D KIDNEY.tif]

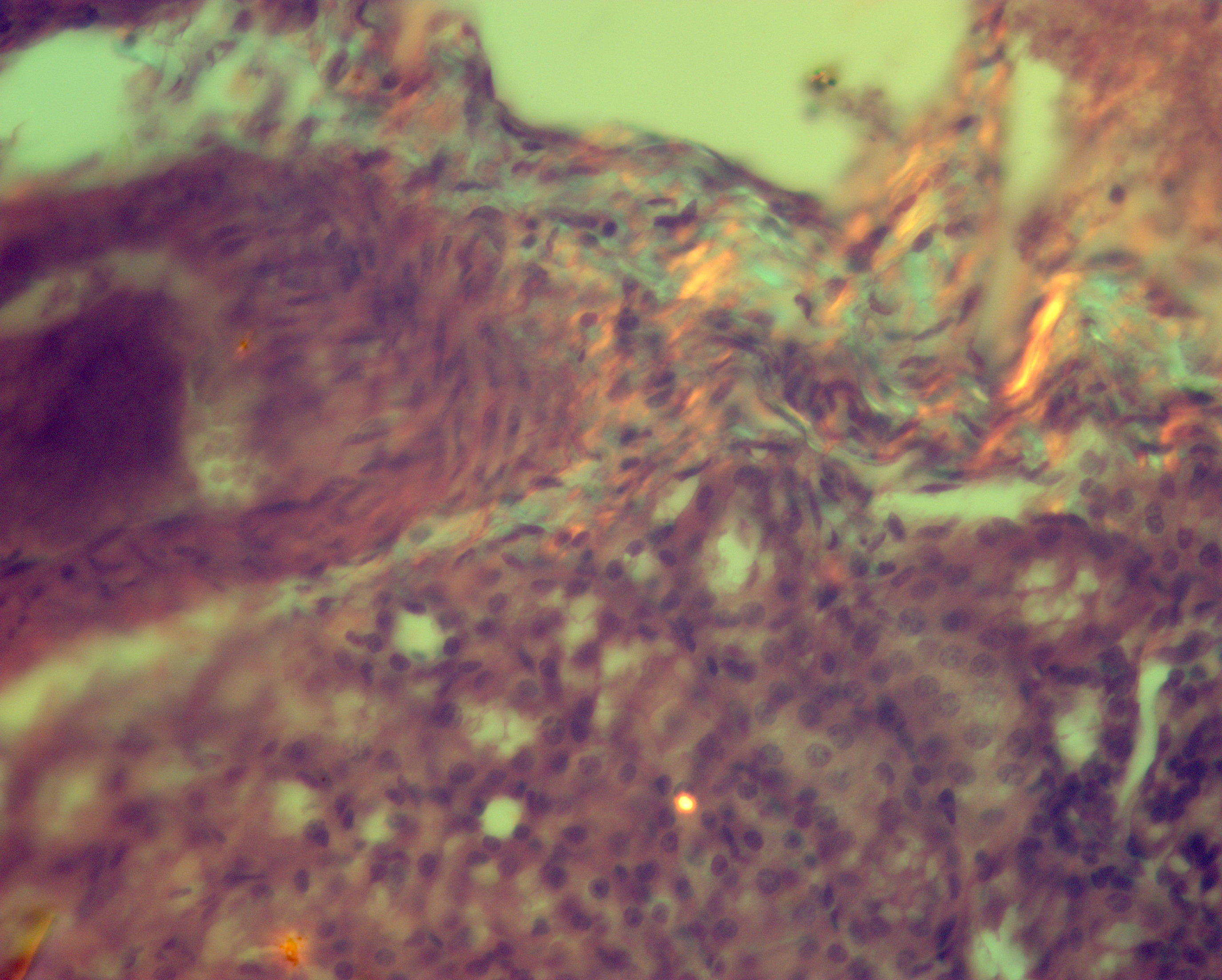

Supplement: Supplementary file 2 — Source data Fig. 1 [file 44321_2024_176_MOESM2_ESM.zip › Figure 1/1K/18D CASEIN KIDNEY.tif]

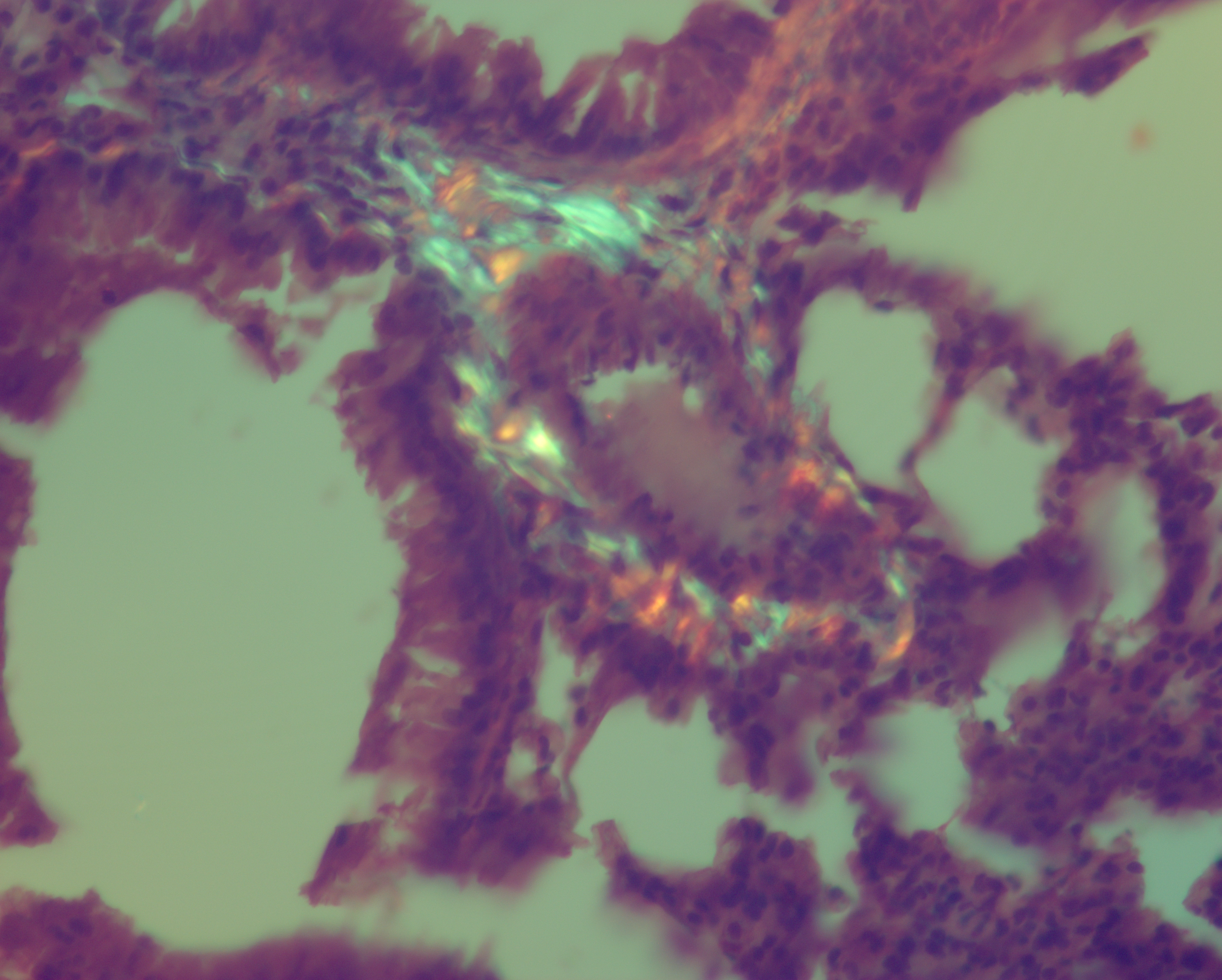

Supplement: Supplementary file 2 — Source data Fig. 1 [file 44321_2024_176_MOESM2_ESM.zip › Figure 1/1K/10D LUNG.tif]

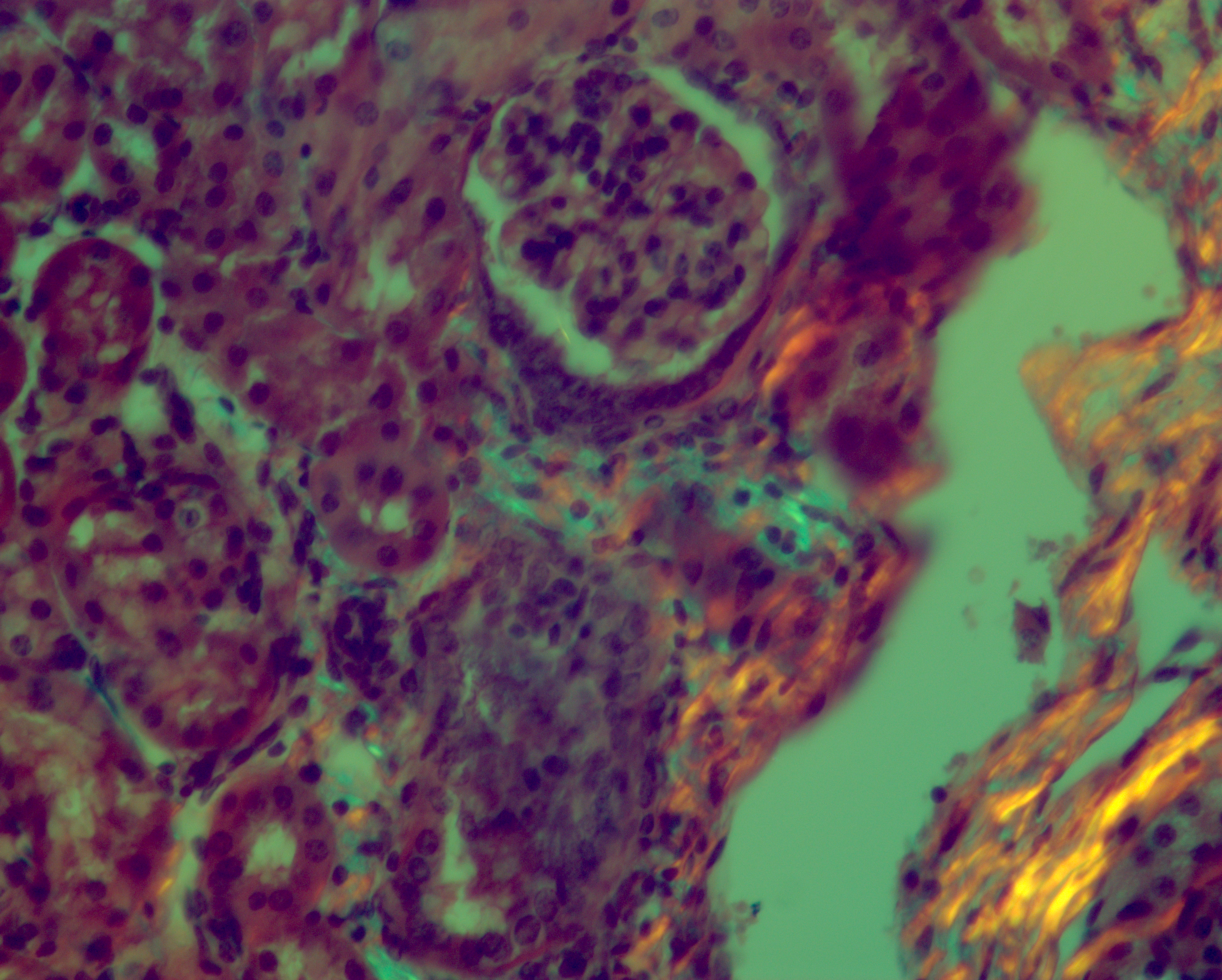

Supplement: Supplementary file 2 — Source data Fig. 1 [file 44321_2024_176_MOESM2_ESM.zip › Figure 1/1K/10D KIDNEY.tif]

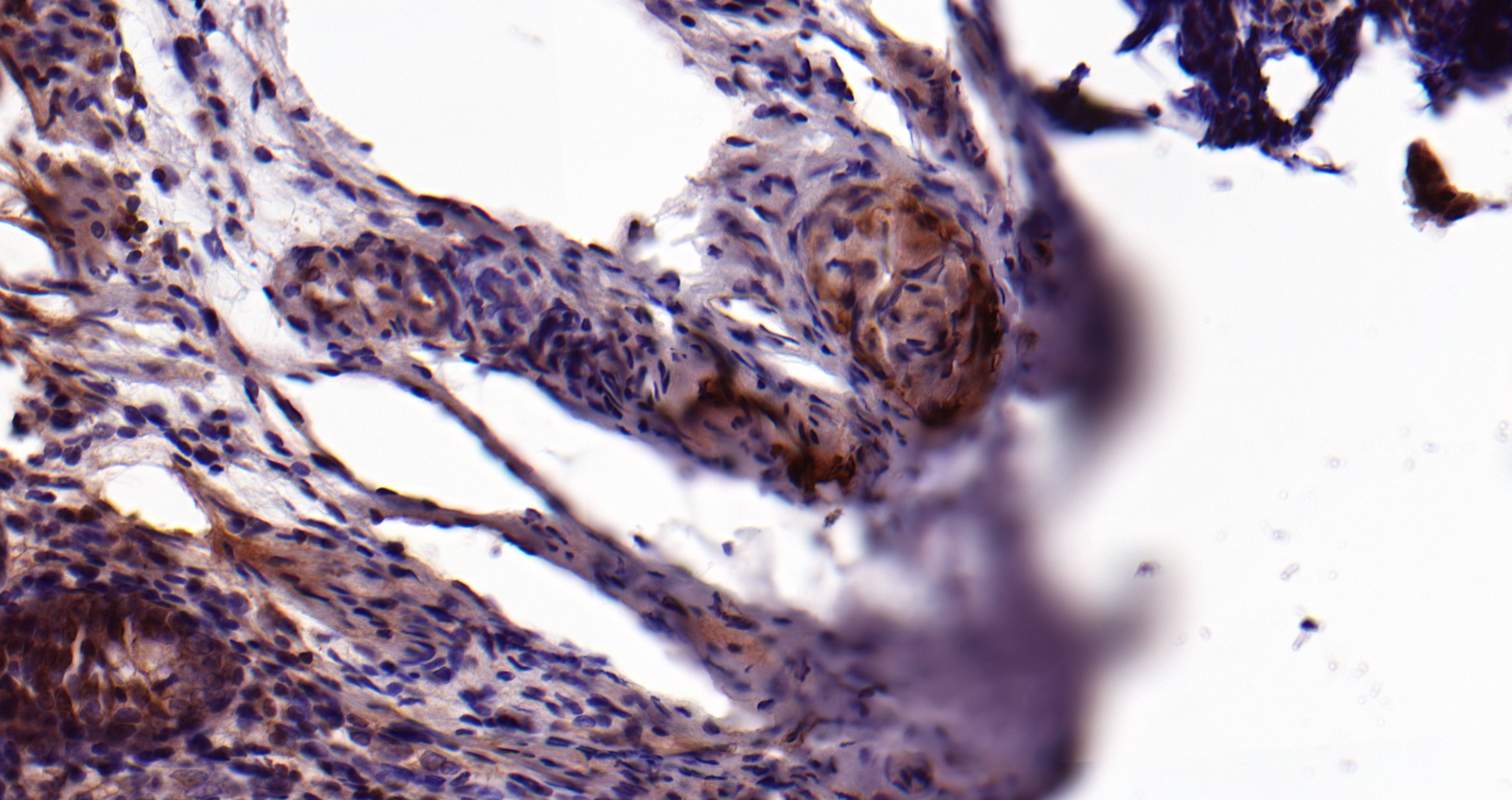

Supplement: Supplementary file 2 — Source data Fig. 1 [file 44321_2024_176_MOESM2_ESM.zip › Figure 1/1B/DUODENUM ASC.tif]

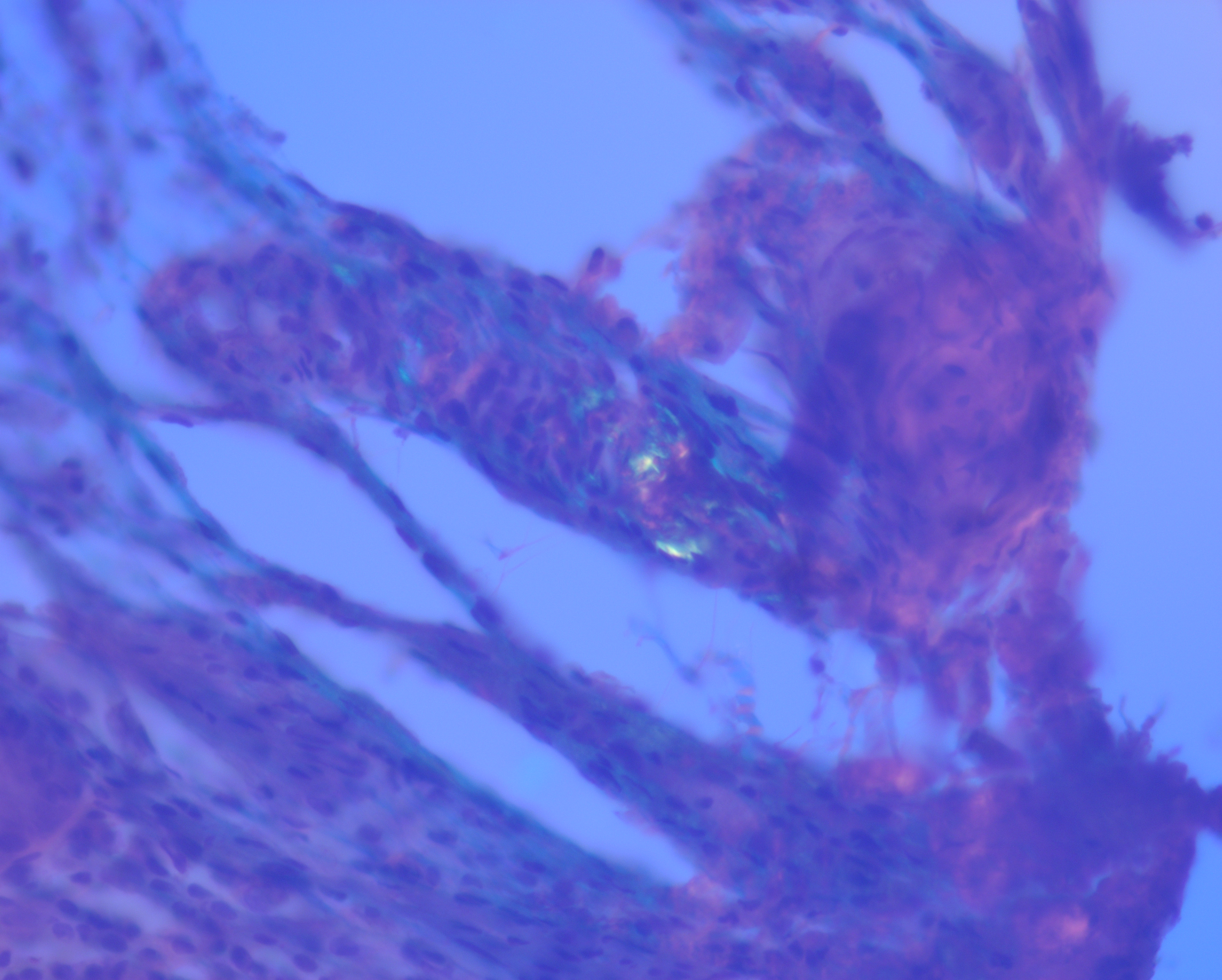

Supplement: Supplementary file 2 — Source data Fig. 1 [file 44321_2024_176_MOESM2_ESM.zip › Figure 1/1B/DUODENUM CONGO RED.tif]
