# Supplementary material for: The inflammasome adaptor protein ASC promotes amyloid deposition in cryopyrin-associated periodic syndromes
Source: EMBO Mol Med. 2024 Dec 5;17(1):41–53. doi: 10.1038/s44321-024-00176-1 (PMC11731034; doi:10.1038/s44321-024-00176-1)
Supplement: Supplementary file 3 — Expanded View Figures [file 44321_2024_176_MOESM3_ESM.pdf]

Expanded View Figures

Figure EV1. NLRP3 p.Q308H resulted in puncta distribution and ASC oligomerization.

(**A**) Representative images of HEK293T cells transfected with NLRP3 wild type (WT) or p.Q308H tagged with YFP; Arrowheads denote cells with a puncta distribution of NLRP3. Scale bars: 10 μm. (**B**) Gating strategy to analyse the percentage of ASC specking cells in five different gates with low constant ASC-RFP expression and increased expression of NLRP3-YFP wild type (top) or p.Q308H (bottom) calculated as mean fluorescence intensity (MFI). NLRP3-YFP MFI corresponding to the five gates are: 1: 300.25; 2: 564; 3: 1063.25; 4: 2023.75; 5: 3693. As example, in the right panels, the percentage of ASC specking cells is shown in the gate number three for NLRP3 expression. (**C**) ASC MFI in human blood lymphocytes (white bar), neutrophils (grey bar) and monocytes (blue bar) cultured for 4 h, from healthy donors and germline CAPS patients whole blood with the NLRP3 variants p.R262W, p.D305N, p.T350M and p.A441T; Centre values represent the mean ($n = 5$ healthy, $n = 5$ patients) and error bars represent s.e.m; one-way ANOVA ($^{\#\#}p = 0.0019$; $^{**}p = 0.0080$; $^{++}p = 0.0040$; *ns*: not significant $p > 0.05$).

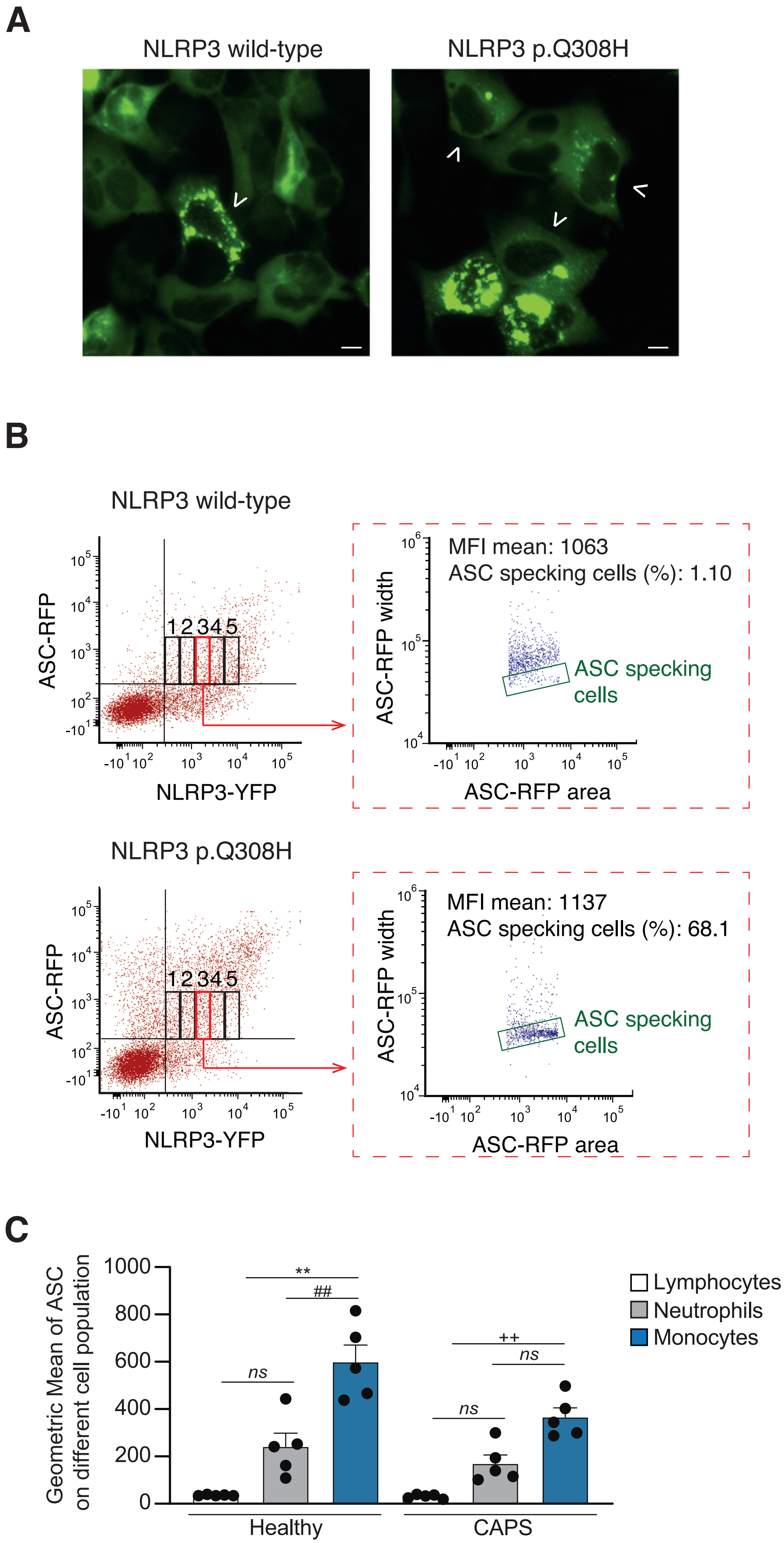
A
NLRP3 wild-type
NLRP3 p.Q308H
B
NLRP3 wild-type
ASC-RFP
NLRP3-YFP
1 2 3 4 5
ASC-RFP width
ASC-RFP area
MFI mean: 1063
ASC specking cells (%): 1.10
ASC specking cells
NLRP3 p.Q308H
MFI mean: 1137
ASC specking cells (%): 68.1
C
Geometric Mean of ASC on different cell population
Lymphocytes
Neutrophils
Monocytes
ns
**
##
++
Healthy
CAPS

 © The Author(s)

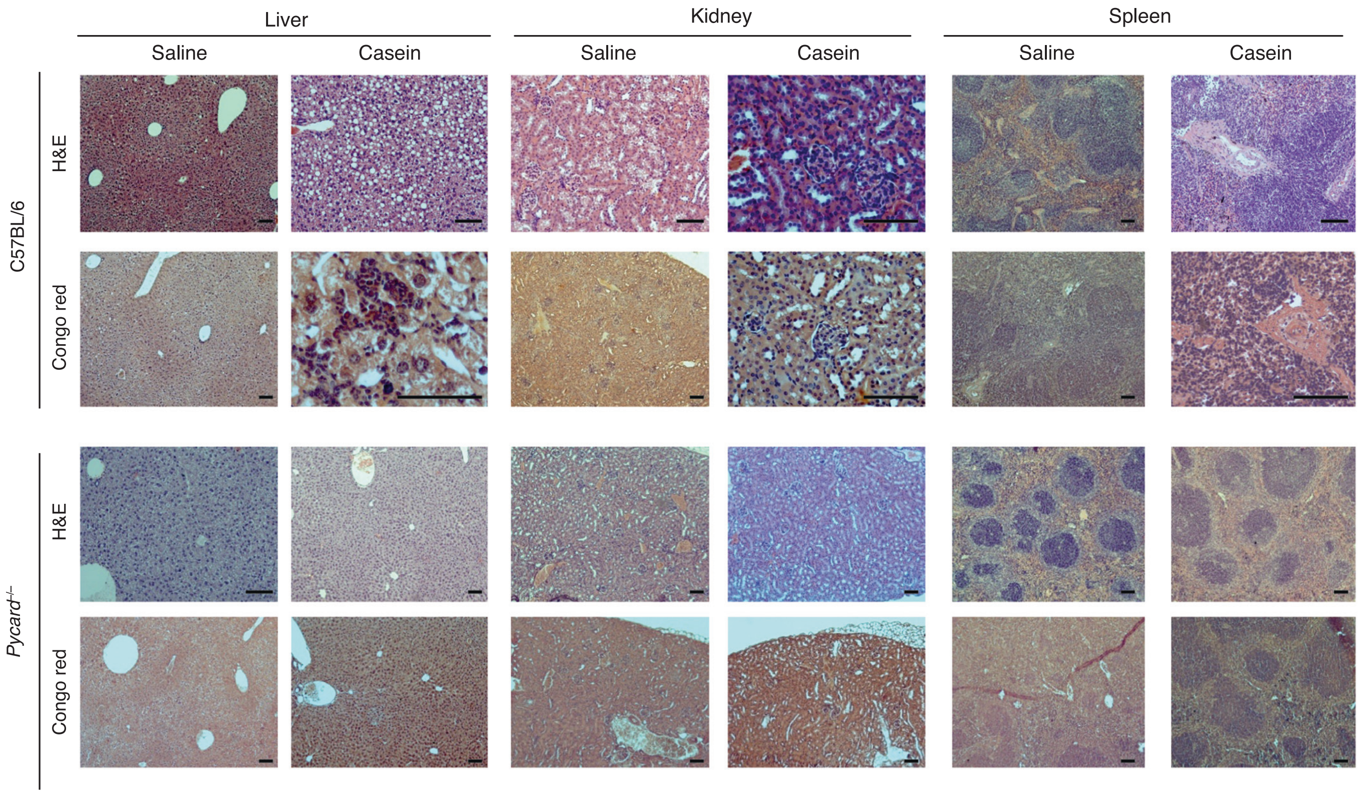

Figure EV2. Amyloidosis is reduced in ASC-deficient mice.

Congo-red and Haematoxylin and eosin stained in liver, kidney and spleen sections from C57BL/6 and *Pycard*$^{-/-}$ mice after 25 days of casein administration. Please note that part of these images are also presented in Fig. 1F. Scale bars: 100 µm.

© The Author(s)

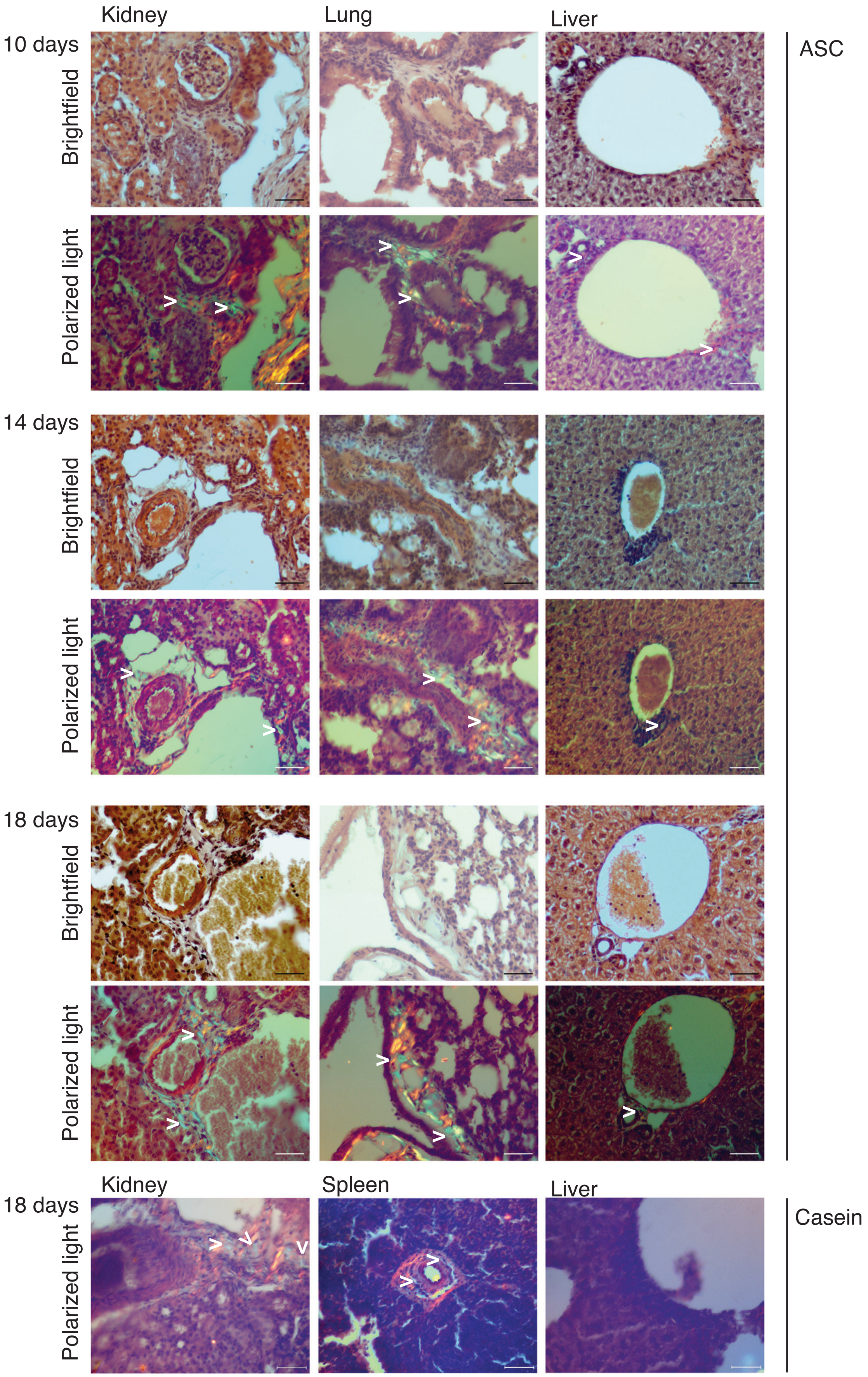
Kidney
Lung
Liver
ASC
10 days
Brightfield
Polarized light
14 days
Brightfield
Polarized light
18 days
Brightfield
Polarized light
Kidney
Spleen
Liver
Casein
18 days
Polarized light

 © The Author(s)

Figure EV3. Administration of ASC oligomers induce amyloidosis in vivo.

Congo-red stained in kidney and lung sections from *Pycard*$^{-/-}$ mice after 10, 14 and 18 days of intraperitoneal ASC oligomers administration. Pictures of brightfield and polarized light are shown. As control, kidney, spleen and liver sections from C57BL/6 mice after 18 days of casein administration were stained with Congo-red and visualized under polarized light. Arrowheads denote areas of amyloid deposition. Please note that part of these images are also presented in Fig. 1J. Scale bars: 50 μm.

© The Author(s)
